# Multi-environment QTL analysis of plant and flower morphological traits in tetraploid rose

**DOI:** 10.1007/s00122-018-3132-4

**Published:** 2018-06-30

**Authors:** Peter M. Bourke, Virginia W. Gitonga, Roeland E. Voorrips, Richard G. F. Visser, Frans A. Krens, Chris Maliepaard

**Affiliations:** 1 Plant Breeding, Wageningen University and Research, Droevendaalsesteeg 1, P.O. Box 386, 6700 AJ Wageningen, The Netherlands; 2 Present Address: Selecta Kenya GmbH & Co. KG, P. O. Box 64132, Nairobi, 00620 Kenya

## Abstract

**Key message:**

Rose morphological traits such as prickles or petal number are influenced by a few key QTL which were detected across different growing environments—necessary for genomics-assisted selection in non-target environments.

**Abstract:**

Rose, one of the world’s most-loved and commercially important ornamental plants, is predominantly tetraploid, possessing four rather than two copies of each chromosome. This condition complicates genetic analysis, and so the majority of previous genetic studies in rose have been performed at the diploid level. However, there may be advantages to performing genetic analyses at the tetraploid level, not least because this is the ploidy level of most breeding germplasm. Here, we apply recently developed methods for quantitative trait loci (QTL) detection in a segregating tetraploid rose population (*F*_1_ = 151) to unravel the genetic control of a number of key morphological traits. These traits were measured both in the Netherlands and Kenya. Since ornamental plant breeding and selection are increasingly being performed at locations other than the production sites, environment-neutral QTL are required to maximise the effectiveness of breeding programmes. We detected a number of robust, multi-environment QTL for such traits as stem and petiole prickles, petal number and stem length that were localised on the recently developed high-density SNP linkage map for rose. Our work explores the complex genetic architecture of these important morphological traits at the tetraploid level, while helping to advance the methods for marker–trait exploration in polyploid species.

**Electronic supplementary material:**

The online version of this article (10.1007/s00122-018-3132-4) contains supplementary material, which is available to authorized users.

## Introduction

Rose (*Rosa x hybrida* L.) is widely considered to be one of the most important ornamental plant species currently cultivated. The genus *Rosa* contains both diploid and tetraploid species with a base chromosome number of seven (2*n* = 4x = 28). A consistent chromosomal numbering scheme has been proposed, based on an integrated consensus linkage map (ICM) that incorporated markers mapped in a number of different rose mapping populations (Spiller et al. 2011). This numbering scheme has also been adopted in the recently published *Rosa chinensis* sequence assembly (Hibrand-Saint Oyant et al. 2018). Consistency in chromosomal numbering has facilitated the comparison of results between studies in rose, helping to confirm the position of important sources of genetic variation for a large number of traits of interest.

Among these traits, understanding the genetic basis of plant and flower morphology in rose has been a major research aim in the rose community for many years. Morphological traits (such as plant vigour, the presence of prickles on stem or petioles, and the number of flower petals) have predominantly been investigated at the diploid level (Crespel et al. 2002; Debener and Mattiesch 1999; Hibrand-Saint Oyant et al. 2008; Li-Marchetti et al. 2017; Roman et al. 2015; Shupert et al. 2005; Yan et al. 2005) with far fewer studies conducted at the tetraploid level (Koning-Boucoiran et al. 2012; Rajapakse et al. 2001). This is no doubt due to the complications of genotype calling, linkage mapping and quantitative trait loci (QTL) analysis in autotetraploids, where methods and software options remain much more limited than for diploids. However, there is an increasing interest in the genetic analysis of autopolyploid species (Bourke et al. 2016, 2017; Hackett et al. 2013, 2017; Schmitz Carley et al. 2017; Voorrips et al. 2011), spurred on by ever-decreasing genotyping costs.

The majority of rose cultivars are tetraploid (Smulders et al. 2011), and most breeding work is performed at the tetraploid level (Gar et al. 2011). Indeed, segregating diploid mapping populations may be developed by the research community, but are of little or no commercial importance to breeders. It has been suggested that this may impose constraints on the size of diploid populations (Debener and Linde 2009). Genetic insights gained at the diploid level are often directly applied to related polyploids, but studies are increasingly identifying genetic and epigenetic control mechanisms at the polyploid level that cannot be directly predicted by progenitor diploid species. Newly formed polyploids may experience homologue loss and genome restructuring, altered patterns of gene expression as well as transcriptional and epigenetic changes (Soltis et al. 2015). For example, in autotetraploid potato, about 10% of a set of 9000 genes were found to be differentially expressed in an experimental autopolyploid series (Stupar et al. 2007). Investigations into inter-allelic interactions are also limited at the diploid level, as there can be at most up to two alleles present in any one individual, and at most four allelic combinations in an *F*_1_ population. By contrast, a tetraploid individual can carry up to four different alleles, and *F*_1_ populations comprise individuals with up to 36 different allelic combinations (ignoring double reduction, which increases this number to 100). Therefore, inter-allelic interactions can only truly be investigated at the polyploid level, preferably in a number of different genetic backgrounds.

As modern cultivated cut rose is probably best classified as a segmental allopolyploid with mainly tetrasomic inheritance (Bourke et al. 2017), it is likely to have been derived from the hybridisation of distinct but closely related progenitor species. As such, insights into both allo- and auto-polyploidisation are potentially relevant. In the well-studied allotetraploid cotton (*Gossypium hirsutum* L.), tetraploids were found to have higher yields and produced a higher-quality fibre than their diploid progenitors grown in the same environment (Jiang et al. 1998). Allohexaploid wheat (*Triticum aestivum*) was found to be significantly more salt tolerant than either its diploid (*Aegiolops tauschii*) or tetraploid (*T. turgidum*) progenitors. This appears to have been a consequence of combining favourable traits from both parents, but also from polyploid-specific phenomena such as the salt-induced expression of a Na^+^ transporter HKT1;5 which was transcriptionally reprogrammed following polyploidisation (Yang et al. 2014). Studies like these emphasise that polyploids may possess emergent properties and traits not found at the diploid level. It therefore appears preferable to investigate important breeding traits at the tetraploid level, both to validate previous studies in diploid rose as well as to understand the genetic control of these traits at the ploidy level at which they are usually selected for.

In this study, we examined a number of morphological traits such as the number of petals, the presence of prickles on stems and petioles, the stem width and length, the chlorophyll content of leaves and the presence of side shoots. These traits were assessed in different locations and were previously the subject of an investigation into possible genotype × environment interactions (Gitonga et al. 2014). Such interactions are of particular relevance in modern-day ornamental breeding, where selection and production are often performed in different locations (Gitonga et al. 2014). Stability of QTL expression over both selection and target environments is needed if marker-assisted selection is to be effectively applied. Here, we re-analyse the data of Gitonga et al. (2014) to perform a QTL analysis using the recently published high-density tetraploid rose linkage map [containing over 25 K single-nucleotide polymorphism (SNP) markers (Bourke et al. 2017)]. Our current study helps to increase our understanding of the genetic control of these traits in tetraploid rose, as well as testing and exploring the effectiveness of recently developed methods for the genetic analysis of autopolyploids.

## Materials and methods

### Plant material and genotyping

**Fig. 1:**
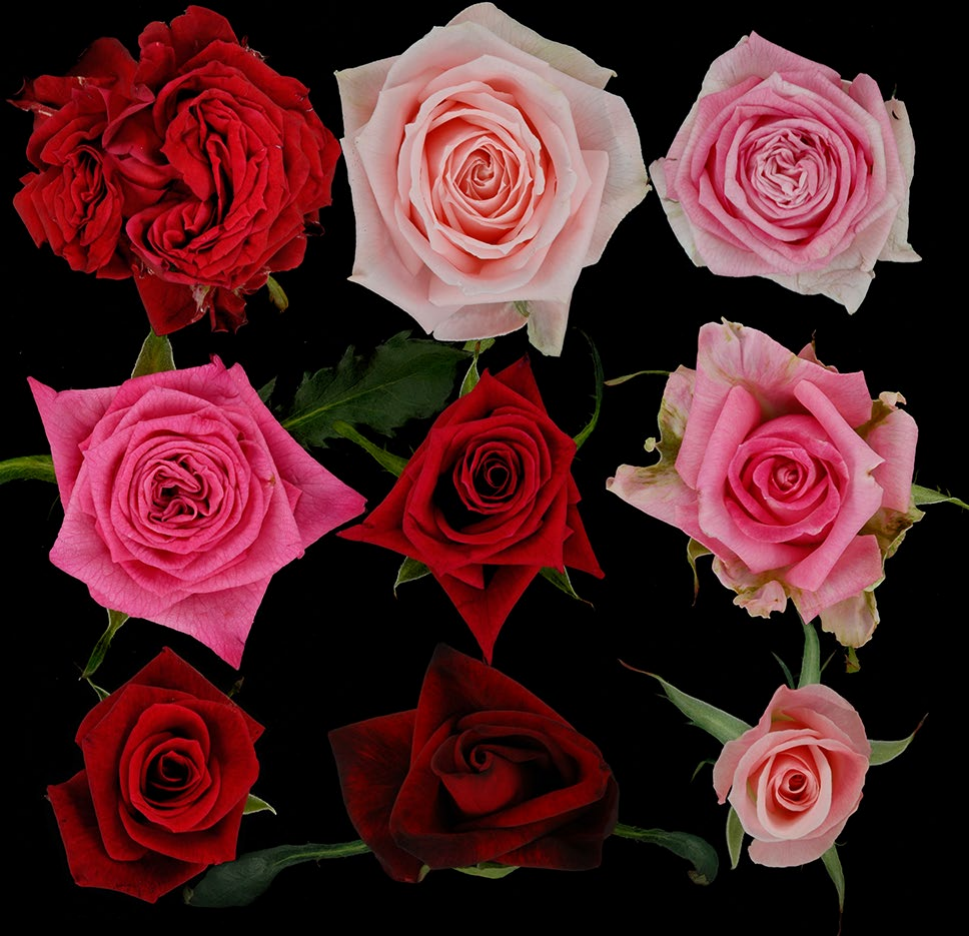
Example of the phenotypic diversity for petal number (and flower colour) in the tetraploid rose K5 population

The tetraploid “K5” rose population, the result of a cross between contrasting lines “P540” and “P867” was used in this study. P540 (the maternal parent) possesses dark red flowers, has prickles on both stem and petiole and is susceptible to powdery mildew, whereas P867 (paternal) has pale salmon-coloured flowers, few to no prickles and is more resistant to powdery mildew (Gitonga et al. 2014; Koning-Boucoiran et al. 2012). The *F*_1_ population resulting from this cross originally comprised of 181 individuals (Yan et al. 2006), but subsequently was found to consist of only 151 unique individuals (Bourke et al. 2017) after genotyping with the 68 K WagRhSNP Axiom SNP array (Koning-Boucoiran et al. 2015). The population segregates for quite a number of important traits [including flower colour, for which a QTL analysis has already been performed (Gitonga et al. 2016)], making it suitable for the current study into morphological traits (an example of the range of phenotypes observed for the number of flower petals is shown in Fig. 1). Discrete dosage calls [ranging from nulliplex condition (dosage = 0) to quadruplex condition (dosage = 4)] were assigned using the fitTetra package in R (Voorrips et al. 2011) as previously described (Bourke et al. 2017).

### Phenotyping

Plant trials were performed at three locations: Wageningen (WAG) in the Netherlands and at Winchester farm, Nairobi (NAI) and Agriflora farm, Njoro (NJO), two production sites in Kenya. In the Netherlands, observations were made during both the summer of 2007 (WAG_S) and the winter of 2007/2008 (WAG_W), whereas in Kenya the observations were made during the period January–July 2009 (using data from the first flowering flush only). Full details of plant propagation and growth conditions are described in Gitonga et al. (2014). A description of the traits measured is provided in Supplementary Table 1. Three juvenile traits (date of bending, plant height and plant vigour) were only recorded in the Wageningen summer trial (WAG_S). All traits were measured with at least two replicates, with 4 individual plants per genotype constituting a replicate. There were two completely randomised blocks for both Wageningen trials, and three completely randomised blocks for both Kenyan trials. Previously, trait heritabilities as well as a comparison of genotype versus genotype × environment variances was performed (Gitonga et al. 2014). Here, we re-calculated best linear unbiased estimates (BLUEs) for traits both within and across environments (single- and multi-environment BLUEs, respectively) using the lmer function of the lme4 package (Bates et al. 2015) in R (R Core Team 2016).

We used the following model for each trait *Y* to estimate single-environment BLUEs:

$$ Y_{ijl} = \mu + G_{i} + \underline{{B_{j} }} + \underline{{\varepsilon_{ijl} }} , $$

where *μ* is the overall mean, *G*_*i*_ is the (fixed) genotype effect, *B*_*j*_ is the (random) block effect, and *ɛ*_*ijl*_ is the (random) residual error.

For the multi-environment BLUEs, we used the following model:

$$ Y_{ijkl} = \mu + G_{i} + \underline{{E_{j} }} + \underline{{B_{jk} }} + \underline{{GE_{ij} }} + \underline{{\varepsilon_{ijkl} }} , $$

where *μ* is the overall mean, *G*_*i*_ is the (fixed) genotype effect, *E*_*j*_ is the (random) effect of environment *j*, *B*_*jk*_ is the (random) effect of block *k* nested within environment *j*, *GE*_*ij*_ is the (random) interaction between genotype *i* and environment *j*, and *ε* is the (random) residual error.

We visualised the consistency between environments through scatter plots of single-environment and multi-environment BLUEs, as well as the frequency distributions of the traits in each of the environments (using the density function in R).

### Linkage map construction and QTL analysis

The linkage map used in the current study has already been published and was created using R scripts developed using previously described methods (Bourke et al. 2016, 2017; Preedy and Hackett 2016). Full details of map construction are described in Bourke et al. (2017). The final integrated linkage maps had 25,695 SNP markers (not all unique positions) and covered 55 of the 56 expected parental homologues (the base chromosome number in *Rosa* is 7; each tetraploid parent is expected therefore to have 28 “homologue” maps, resulting in 56 maps across both parents).

A subset of these markers was chosen for the estimation of inheritance probabilities in the population using the TetraOrigin software (Zheng et al. 2016). In an outcrossing autotetraploid, there are nine distinct marker segregation types, namely 1 × 0, 0 × 1, 2 × 0, 0 × 2, 1 × 1, 1 × 3, 1 × 2, 2 × 1 and 2 × 2, where the numbers represent the dosage of the marker in the mother and father, respectively. All other marker types (e.g. 4 × 1) can be converted to one of these 9 types without loss or distortion of their linkage information (Bourke et al. 2016). For each 1-centiMorgan (cM) interval, a single marker from each marker segregation type was selected (if possible) which had the lowest number of missing observations across the population. TetraOrigin (Zheng et al. 2016) was run on Mathematica version 10 (Wolfram Research Inc. 2014), allowing both bivalent_decoding options (False/True) in the ancestral inference stage. This generated identity-by-descent (IBD) probabilities for the population under a model that allowed for both bivalents and multivalents in the parental meiosis (i.e. bivalent_decoding = False), as well as a purely bivalent model for which double reduction is ignored (i.e. bivalent_decoding = True). In the latter case, unexpected scores are treated as genotyping errors by the software. The following settings were used: parental dosage error probability (epsF) = 0; offspring dosage error probability (eps) = 0.001; parental bivalentPhasing = True (which assumes that bivalent pairing predominates across the population in the determination of parental marker phase). The IBD probabilities at the marker positions were interpolated at 1-cM intervals using the default settings of smooth.spline in R (R Core Team 2016) and saved for subsequent QTL analysis.

We compared both a one-stage and two-stage analysis to detect QTL. For the one-stage analysis, we converted the 36-genotype probabilities into 8 haplotype probabilities (these are also provided in the TetraOrigin output), where each haplotype probability *H*_*i*_ is the inheritance probability of haplotype *i* at a particular locus per individual. We first fit the “full” model using the lmer function from the lme4 package (Bates et al. 2015) with option REML = FALSE (i.e. using maximum likelihood) as follows:

$$ Y_{jkl} = \mu + H_{2} + H_{3} + H_{4} + H_{6} + H_{7} + H_{8} + \underline{{E_{j} }} + \underline{{B_{jk} }} + \underline{{\varepsilon_{jkl} }} , $$

where the fixed-effect terms *H*_*1*_ and *H*_*5*_ were dropped to satisfy the boundary conditions $\sum\nolimits_{i = 1}^{4} {H_{i} = 2}$ and $\sum\nolimits_{i = 5}^{8} {H_{i} = 2}$ (and where *μ* is the intercept term). The random part of the model containing environmental (*E*) and block (*B*) effects was also separately fit using the lmer function as follows:

$$ Y_{jkl} = \mu + \underline{{E_{j} }} + \underline{{B_{jk} }} + \underline{{\varepsilon_{jkl} }} . $$

We performed a model comparison using the anova function in R, which performs a likelihood ratio test and returns a *p* value from a comparison of the test statistic to a χ^2^ distribution. The − log_10_(*p*) values therefore give an approximation to the more usual LOD scores from similar genetic studies. However, we also wanted to determine empirical significance thresholds, for which we ran permutation tests (Churchill and Doerge 1994) with 1000 permutations and *α* = 0.05, permuting the order of the haplotype probabilities and saving the maximum − log_10_(*p*) value from each genome-wide scan to generate approximate 95% genome-wide significance thresholds.

The alternative approach (“two-stage analysis”) we tested was a weighted linear regression on the single-environment BLUEs, with the 36-genotype IBD probabilities as weights. We first fit environmental effects (*E*) in the following simple linear model:

$$ Y_{jk} = \mu + E_{j} + \varepsilon_{jk} . $$

This allowed us to impute any missing observations using the fitted model (but only in cases of 1 missing value—we did not impute if 2 or more of the 4 observations were missing). The residuals *ɛ*_*jk*_ from this were carried forward as the *Y* vector to subsequently detect QTL effects. The form of the model used has been described in detail elsewhere (Hackett et al. 2013, 2014; Kempthorne 1957), namely:

$$ Y = \mu + \alpha_{2} X_{2} + \alpha_{3} X_{3} + \alpha_{4} X_{4} + \alpha_{6} X_{6} + \alpha_{7} X_{7} + \alpha_{8} X_{8} + \varepsilon , $$

where each *X*_*i*_ is an indicator variable for one of the eight parental homologues, having taken the inheritance constraints $\sum\nolimits_{i = 1}^{4} {X_{i} = 2}$ and $\sum\nolimits_{i = 5}^{8} {X_{i} = 2}$ into account, and weighting by the IBD probabilities as calculated by TetraOrigin (and *μ* is the intercept). Note that because of the balanced design, this is equivalent to including environment as a fixed term in the QTL model (although our approach also allowed the inclusion of incomplete data within an ordinary linear model context).

For the traits bending time, height and vigour that were measured in the Wageningen summer season alone (WAG_S), a single-environment analysis was performed, using the phenotype values rather than residuals as the dependent variable. Genome-wide significance thresholds per trait were determined using permutation tests by recording the maximum LOD score from each of 1000 genome-wide QTL scans using permuted genotypes, with the 95th percentile of the sorted LOD scores taken as the threshold. Single-environment analyses were also performed to assess the stability of QTL across environments, with significance thresholds per environment and per trait determined using permutation tests as described. To facilitate visualisation, we plotted LOD profiles from the single- and multi-environment analyses together, with the single-environment profiles adjusted so that significance thresholds were equal (i.e. with multiple y-axes on a single plot). Regions for which the LOD profile of the multi-environment QTL analysis exceeded the significance threshold were re-mapped by saturating the LOD-2 intervals of the QTL peaks with extra markers before re-running TetraOrigin to generate more precise IBD probabilities in the vicinity of QTL. These extra markers were selected as previously described but with a binning window of 0.1 cM in the QTL interval, added to the already selected marker set from the initial QTL scan. We used the two-stage approach for QTL detection (weighted regression on the single-environment BLUEs, with the IBD probabilities as weights), focusing on the linkage groups where QTL were originally detected.

QTL peaks from the (marker-saturated) multi-environment analysis were also explored to determine the most likely QTL segregation type and mode of action using the Bayesian information criterion (BIC) (Schwarz 1978) as described by Hackett et al. (2013, 2014). We tested all bi-allelic additive, simplex-dominant and duplex-dominant configurations [where simplex- and duplex-dominant are defined by the number of alleles required to give full expression of the QTL (either 1 or 2 copies, respectively) (Rosyara et al. 2016)]. We also tested for multi-allelic QTL by considering all possible configurations of up to five different alleles, with unconstrained allele effects [so that for example three alleles *Q*_1_, *Q*_4_ and *Q*_8_ offspring QTL classes—*Q*_1_, *Q*_4_, *Q*_8_, *Q*_1_*Q*_4_, *Q*_1_*Q*_8_, *Q*_4_*Q*_8_ and *Q*_1_*Q*_4_*Q*_8_ were assumed to have different means. This is equivalent to the “codominant factor” designation in TetraploidSNPMap (Hackett et al. 2017)]. We estimated the average contribution of each homologue as $\overline{h} - \overline{y}$ where $\overline{h} = \left( {\sum\nolimits_{i = 1}^{N} {\pi_{i} y_{i} } } \right)/\left( {\sum\nolimits_{i = 1}^{N} {\pi_{i} } } \right)$ using IBD probabilities *π*_*i*_, multi-environment BLUEs *y*_*i*_ and overall population mean $\overline{y}$. These were visualised to help clarify the allelic effects around QTL peaks. The average QTL allele effect was estimated using a weighted regression of the QTL genotype counts on the 36 genotype means, using the cumulative probability in each of the 36 genotype classes as weights (i.e. weighted by the approximate number of individuals in each class, which sum to *N*). The slope of the regression and standard error of the estimate were recorded. In the case of multi-allelic QTL, the effect of each allele was estimated separately. For QTL predicted to exhibit dominance, QTL genotype counts were coded as 0 or 1 for both simplex and duplex dominance models (with a 1 being assigned to QTL genotype classes carrying at least 1 (respectively 2) copies of the predicted QTL alleles for simplex (respectively duplex) dominant QTL).

For comparison purposes, we also conducted a single-marker analysis of variance (ANOVA) for each trait on the marker dosage classes for all mapped markers (using the multi-environment BLUEs as phenotypes), with the − log_10_(*p* value) of the model fit used as a proxy for the LOD score. Significance thresholds were determined using permutation tests with *N* = 1000 and *α* = 0.05 as described above. ANOVA and IBD-based results were visualised together to enable a comparison of the two approaches, adjusted so that significance thresholds overlapped.

A genetic co-factor analysis was performed using the detected QTL peak positions as co-factors in a two-stage analysis as previously described. In cases where multiple QTL were found, all QTL were simultaneously used as co-factors within a single model. Each QTL was supplied as a set of six fixed terms (*H2*, *H3*, *H4*, *H6*, *H7* and *H8* corresponding to six of the eight parental haplotype probabilities at the QTL peak for the population, accounting for the inheritance dependence between them). Significance thresholds were re-calculated using permutation tests.

The genotypic information coefficient (GIC) per homologue, a measure of how much information we have to estimate QTL effects across the mapped genome, was determined using the following formula:

$$ {\text{GIC}}_{j} = 1 - \frac{4}{N}\mathop \sum \limits_{n = 1}^{N} \pi \left( {1 - \pi } \right) , $$

where *π* is the inheritance probability of homologue *j* in individual *n* at a particular locus, estimated using TetraOrigin (Bourke et al. 2018).

## Results

### One-stage versus two-stage QTL analysis

**Fig. 2:**
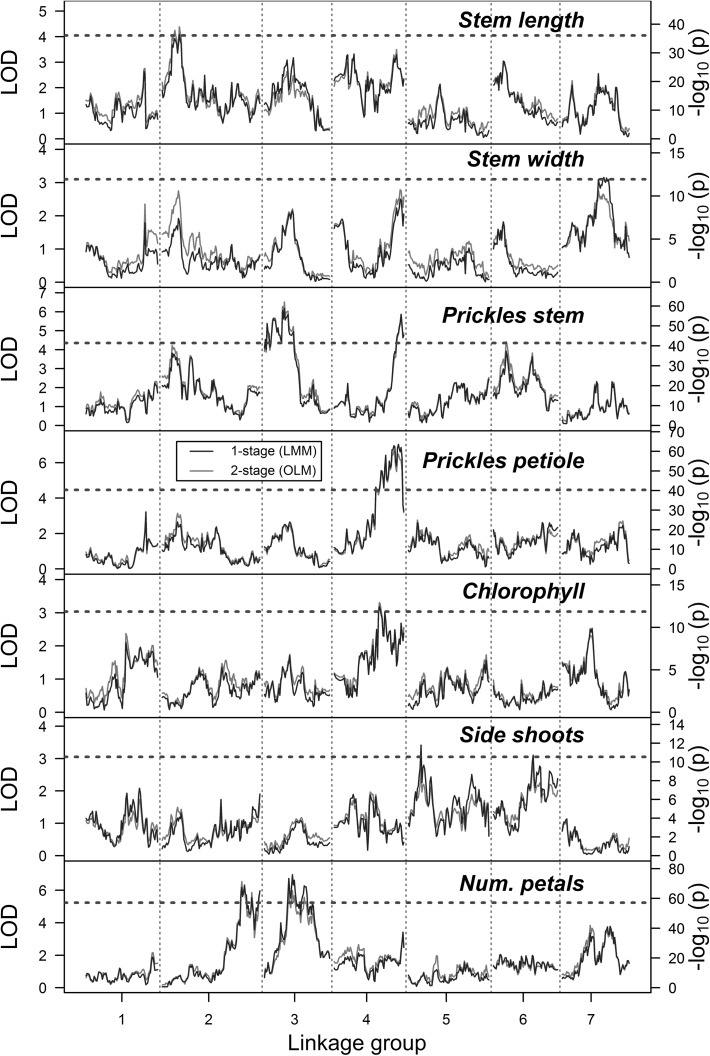
Comparison of the QTL results using a one-stage linear mixed model approach versus a two-stage analysis using BLUEs in an ordinary linear model. Significance thresholds, as determined by permutation tests (*N* = 1000, *α* = 0.05), are shown as dashed red lines (data were re-scaled so that these overlap). The one-stage results (− log_10_(*p*) values) are shown in blue, with the two-stage results (LOD scores) shown in red (color figure online)

In this study, we compared a one-stage mixed linear model analysis versus a two-stage approach which used mixed models only in the first stage to estimate BLUEs and used a weighted linear regression in the second stage to detect QTL effects. Our two-stage procedure is a simplification of a more formal two-stage analysis that carries variance estimates as well as BLUEs forward to a second-stage mixed model analysis (Damesa et al. 2017; Möhring and Piepho 2009). Overall, the results of both approaches were very similar (Fig. 2). The one-stage approach was arguably more powerful, detecting two QTL for side shoots which were not picked up in the two-stage analysis (just falling short of significance) as well as a QTL for stem width.

### Single- versus multi-environment analyses

**Fig. 3:**
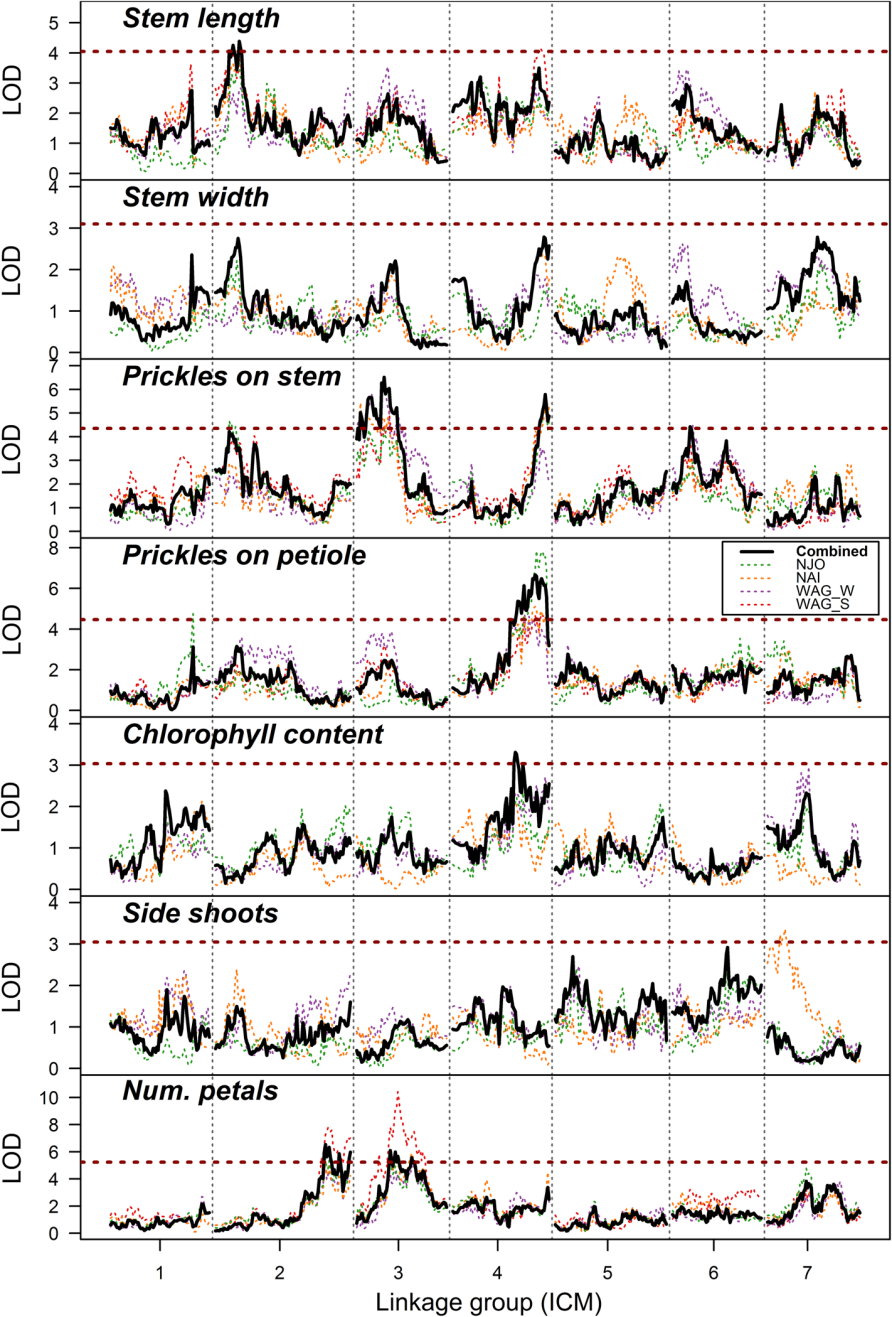
LOD profiles of QTL scans for morphological traits across different growing environments in the K5 tetraploid rose population using the two-stage approach. The results from single-environment analyses are shown as coloured dashed lines, with the multi-environment analysis results as a solid black line (“Combined”). Environments tested were Njoro (“NJO”), Nairobi (“NAI”), Wageningen winter (“WAG_W”) and Wageningen summer (“WAG_S”). Significance thresholds for the multi-environment analysis, as determined by permutation tests, are shown as red dashed lines. Single-environment LOD profiles (dashed QTL lines) were re-scaled so that the significance thresholds of all plots coincided precisely, i.e. the y-axis LOD scales are only correct for the combined analysis (solid black line) (color figure online)

We discovered a number of QTL in this study, although not all traits produced significant peaks (Figs. 2, 3). Table 1 summarises the main QTL findings from the initial QTL scan as well as those from the subsequent exploration of the QTL peaks. For most traits, the QTL position, LOD score and explained variances were relatively consistent between the initial QTL scan and the subsequent re-mapping. Some minor QTL were detected in single-environment analyses that were not confirmed in the multi-environment analysis [e.g. the QTL for stem length on ICM 4 detected in the Wageningen summer trial, or the QTL for stem prickles on ICM 2 detected in Njoro (Fig. 3)]. However, on the whole we found that single-environment and multi-environment analyses corresponded quite well. When we examined the trait values, we found that they were relatively consistent across environments (Supplementary Fig. 1). However, for the traits chlorophyll content and side shoots, we observed a poor correlation across environments, with correlation coefficients as low as *R*^2^ = 0.52 in the case of chlorophyll content recorded in Nairobi when compared to the multi-environment BLUEs. The frequency distributions of the phenotypes across each environment showed that some traits (like the number of petals) had a very similar distribution of values across environments, whereas other (like chlorophyll content) did not (Supplementary Fig. 2).

**Table 1:** Summary of multi-environment QTL detected in this study using two-stage QTL analysis

Trait	N^a^	Thresh.^b^	ICM^c^	cM^d^	LOD^e^	Expl. Var^f^	Model^g^	Act.^h^	Dir.^i^	Effect^j^	SE^k^
Stem length	121	4.0	2	19 (19.8)^l^	4.4 (4.6)^l^	0.14 (0.15)^l^	oQ_2_Q_3_o × ooQ_7_Q_8_	CD	—	****	****
Stem prickles	121	4.4	3	22 (27.4)	6.5 (7.8)	0.21 (0.25)	ooQQ × Qooo	SD	+	2.75	0.67
			4	74 (74.5)	5.8 (6.1)	0.19 (0.20)	oooQ × oQoo	A	−	1.92	0.34
			6	14 (14.4)	4.4 (4.1)	0.14 (0.13)	QQoQ × oQoo	A	−	1.61	0.32
Petiole prickles	121	4.5	4	66 (64.7)	6.7 (7.0)	0.21 (0.22)	QQoo × Qooo	A	+	0.78	0.12
Num. petals	120	5.2	2	88 (92.7)	6.5 (7.1)	0.21 (0.23)	QooQ × oooQ	A	+	8.62	1.46
			3	27 (33.7)	6.1 (6.6)	0.20 (0.21)	oQQo × oooQ	DD	−	6.71	1.01
Chlorophyll	121	3.0	4	50 (50.3)	3.3 (3.5)	0.11 (0.11)	oQQo × QQoo	SD	+	2.95	0.95

^a^*N* number of offspring for which genotype and phenotype data were available for each trait

^b^*Thresh.* experiment-wide LOD significance threshold, determined by permutation test with *N* = 1000 and *α* = 0.05

^c^*ICM* chromosomal linkage group, using the integrated consensus map (ICM) numbering of Spiller et al. (2011) and Bourke et al. (2017)

^d^*cM* centiMorgan position of QTL peak

^e^*LOD* logarithm of odds at QTL peak

^f^*Expl. Var.* fraction of phenotypic variance explained by the QTL model at the peak position ($R_{\text{adj}}^{2}$ of the linear model)

^g^*Model* QTL model that best fit the data at the QTL position (i.e. minimised the BIC). “*Q*” signifies a predicted QTL allele with an estimated effect, whereas “*o*” denotes an allele with neutral effect (i.e. the remaining, grouped alleles at a QTL whose effects are not estimated). In cases where the most likely model was multi-allelic, subscripts (*Q*_1_ etc.) are used to denote the parental origin of the allele. Note that in the case of dominance, the *Q* (or* QQ*) alleles are taken to be dominant

^h^*Act.* mode of action of QTL model, *A* (bi-allelic) additive, *CD* (multi-allelic) codominant, *SD* (bi-allelic) simplex dominant, *DD* (bi-allelic) duplex dominant

^i^*Dir.* Direction of the allele effect, either positive (+), i.e. increasing trait value, or negative (−). In the case of multiple QTL alleles, the direction of each QTL allele *Q*_*n*_ is given

^j^*Effect* QTL allele effect, estimated using the slope of a (weighted) regression of the genotype means from the 36 genotype classes against the QTL allele count (coded as 0/1 in the case of both simplex or duplex dominance to signify the absence or presence of the dominance-causing alleles)

^k^*SE* standard error of the estimated slope of the regression line

^l^Numbers shown are results from initial QTL scan, with those in parentheses the results following re-saturation of QTL region with additional markers

****Q_2_ = 1.28 ± 1.76, *Q*_3_ = 2.35 ± 1.73, *Q*_7_ = 2.78 ± 1.73, *Q*_8_ = 5.87 ± 1.47

### Determining QTL allelic composition

**Fig. 4:**
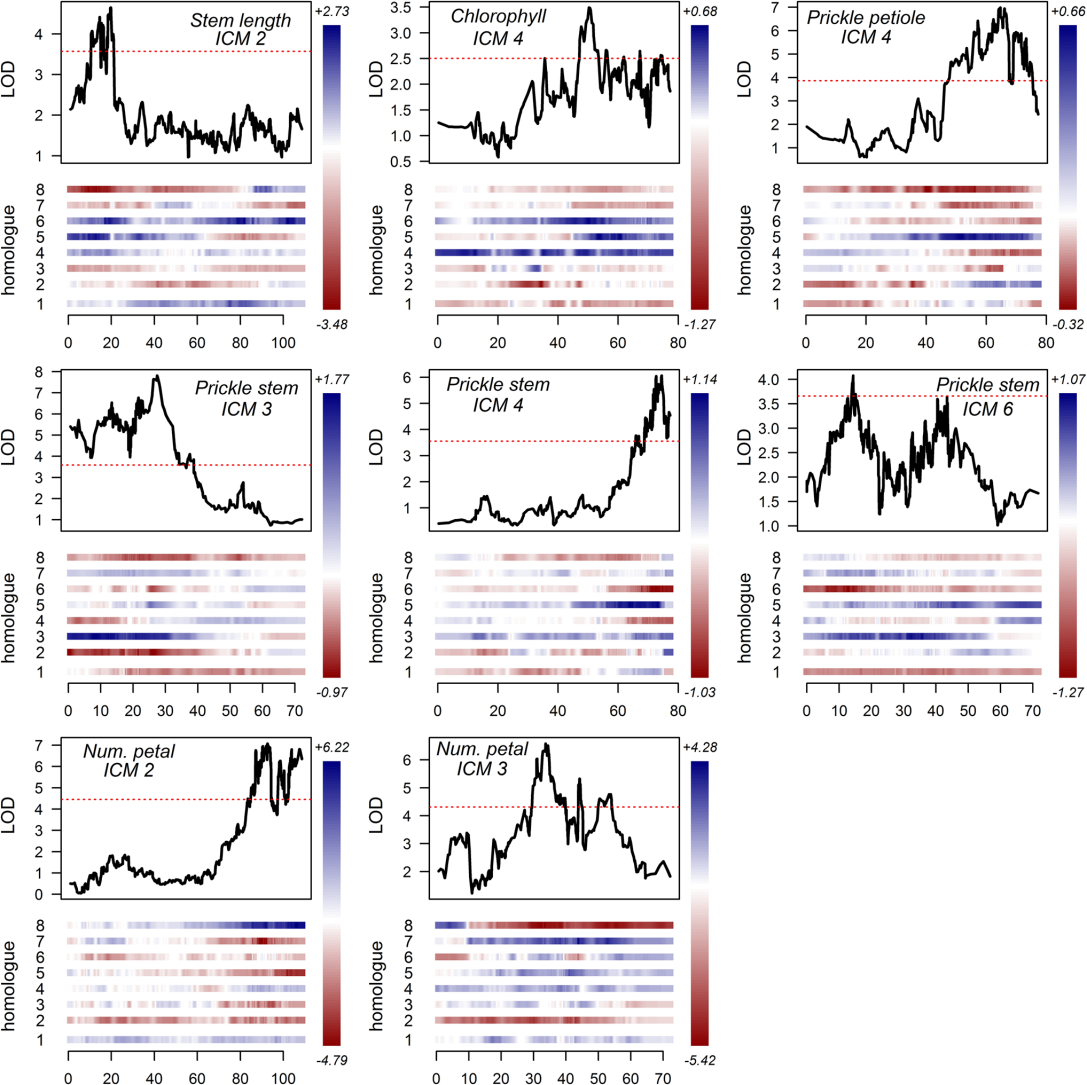
Allelic effects around QTL peaks detected for the morphological traits stem length, chlorophyll content, prickles on petiole and stem, and number of petals. For each QTL peak, the IBD-weighted phenotype mean contribution of each parental homologue (1–4 for parent 1 and 5–8 for parent 2) are shown below the LOD profile, with darker blue representing a positive influence on the trait, and red a negative influence. LOD significance thresholds as determined by permutation tests (*N* = 1000, *α* = 0.05) are shown as dotted red lines. The range of allele effects (+/−) is shown above and below the scale to the right of each plot. LOD profiles correspond to a two-stage QTL analysis using single-environment BLUEs as described in the main text. Chromosome numbering (ICM) is according to the integrated consensus map numbering of Spiller et al. (2011) (color figure online)

Our analysis of the most likely allelic configurations showed that at least one QTL peak was likely to be influenced by multiple alleles, with various models of gene action predicted (Table 1). We visualised the allele effects across the chromosomes harbouring QTL, revealing the multi-allelic nature of many of these loci (Fig. 4). However, the prediction of the best-fitting QTL model using the BIC procedure was far from clear, as for all of the traits there were more than one model within 6 BIC of the minima, i.e. no clear QTL model front-runners were identified (we only report the most likely model in Table 1). Most of the QTL had relatively modest effect sizes, although the positive QTL allele on ICM 2 for petal number contributed approximately 8.6 ± 1.5 extra petals (Table 1). Incorporating double reduction into the QTL model did not result in any significant differences to the results, and therefore for simplicity we have chosen only to report the results from an analysis assuming random bivalent pairing. For the juvenile traits bending time, plant height and vigour, we found two QTL for height on ICM 2 and 6, with no QTL for the other two traits (Supplementary Fig. 3). However, since we only had phenotypic data for these traits in a single environment (WAG_S), we were unable to make any prediction about the robustness of these QTL across environments.

### Single-marker ANOVA

The single-marker ANOVA analysis on the seven multi-environment morphological traits in this study produced slightly different results to that using the IBD probabilities (Table 2; Supplementary Fig. 4). In some cases, the power of QTL detection was slightly higher using the ANOVA approach; for example, we detected a second peak for chlorophyll content on linkage group ICM 7 which was not detected using the IBD model (although there was a non-significant peak at the same position). A single QTL for stem width was identified on ICM 7 with the ANOVA model, which we found using the one-stage procedure but not the two-stage procedure. On the whole though, the results from the single-marker ANOVA analysis very much confirmed the results we found using IBD probabilities.

**Table 2:** Summary of multi-environment QTL detected in this study using the single-marker ANOVA additive effects model

Trait	N^a^	Threshold^b^	ICM^c^	cM^d^	− log_10_(p)^e^	Expl. Var^f^	Peak marker^g^	Marker phase^h^
Stem length	121	4.9	2	14.6	5.1	0.15	M36499_924	oooo × QQoo
Stem width	121	4.7	7	47.6	6.0	0.18	K3954_219	oooQ × oooo
Prickles on stem	121	4.8	3	21.4	7.4	0.22	K7826_576	ooQo × ooQo
			4	72.2	6.7	0.20	K5629_995	SxS
Prickles on petiole	121	4.8	4	66.9	7.7	0.24	G38418_730	QooQ × oQQo
Chlorophyll content	121	4.9	4	45.9	5.9	0.19	K5520_777	oooQ × oQoo
Num. petals	121	4.8	2	98.3	6.6	0.20	G66895_409	oooo × ooQo
			3	31.5	6.4	0.19	K599_2377	ooQo × ooQo

^a^*N* number of offspring for which genotype and phenotype data were available for each trait

^b^*Threshold* experiment-wide significance threshold, determined by permutation test with *N* = 1000 and *α* = 0.05

^c^*ICM* chromosomal linkage group, using the integrated consensus map (ICM) numbering of Spiller et al. (2011) and Bourke et al. (2017)

^d^*cM* centiMorgan position of QTL peak

^e^ − log_10_(p) = significance at QTL peak, using the p value from the model fit

^f^*Expl. Var.* fraction of phenotypic variance explained by the QTL model at the peak position (adjusted *R*^2^ from the regression)

^g^*Peak marker* marker with the highest trait association above the threshold on that linkage group

^h^*Marker phase* parental marker phase (consistent with homologue numbering from Table 1 and Fig. 4). In cases where the marker was not phased due to insufficient linkage to 1 × 0 markers, the segregation type is given instead

### Comparison with previously reported QTL

A comparison of our results with those of previous studies showed that some QTL had already been identified in other populations on the same linkage groups as we identified, while some QTL differed (Table 3). For example, an earlier study in the diploid 94/1 population found a QTL for both stem length and width on ICM 2 (Yan et al. 2007), although they also identified QTL on ICM 1 and 5 for these traits which we did not. For the trait stem prickles, we identified QTL on ICM 3, 4 and 6, whereas previously stem prickle QTL have been detected on ICM 2, 3 and 4 (Crespel et al. 2002; Koning-Boucoiran et al. 2012; Linde et al. 2006). The QTL on ICM 2 as reported by Koning-Boucoiran et al. (2012) was found using the same K5 mapping population (and same phenotype data) as here. However, in our analysis the peak on ICM 2 fell just below the significance threshold (Fig. 3), and indeed was detected when the analysis was restricted to Njoro data only.

**Table 3:** Overview of previous QTL detected for the morphological traits in this study

Trait	Population	N^a^	Ploidy^b^	LG^c^	References
Stem length	94/1	88	2x	1	
				2	
				5	Yan et al. (2007)
Stem width	94/1	88	2x	1	
				2	
				5	Yan et al. (2007)
Stem prickles	K5	184	4x	2	
				3	Koning-Boucoiran et al. (2012)
	HW	91	2x	4	Crespel et al. (2002)
	97/7	270	2x	3	Linde et al. (2006)
Petiole prickles	90-69 F_2_	52	4x	6^d^	Rajapakse et al. (2001)
	90-69 F_2_	52	4x	6^d^	Zhang et al. (2006)
Chlorophyll content	94/1	88	2x	2	
				3	
				6	
				7	Yan et al. (2007)
Num. petals	94/1	60	2x	3	Debener and Mattiesch (1999)
	HW	91	2x	6	Crespel et al. (2002)
	97/7	270	2x	3	Linde et al. (2006)
	HW	91	2x	4	Hibrand-Saint Oyant et al. (2008)
	K5	184	4x	3	Koning-Boucoiran et al. (2012)

^a^*N* mapping population size used

^b^Ploidy of mapping population used, either diploid (2x) or tetraploid (4x)

^c^*LG* linkage group numbering according to the integrated consensus map (ICM) numbering of Spiller et al. (2011)

^d^In the case of the Rajapakse et al. (2001) and Zhang et al. (2006) studies, the numbering was imputed through linkage with microsatellite markers (see main text for details)

### Co-factor analysis

We performed a simple genetic co-factor analysis by including all detected QTL as genetic covariates in a subsequent re-analysis. In most cases, the inclusion of detected QTL had little or no impact on the QTL results (Supplementary Fig. 5). However, in the case of stem length we picked up a novel QTL on linkage group ICM 4 (66 cM, LOD = 4.3, $R_{\text{adj}}^{2} \, = \,0.14$). This corresponded closely to the position of a single-environment QTL for stem length detected in the WAG_S trial (Fig. 3). For the trait number of petals, there was also a marked increase in significance of an ICM 4 position in the co-factor analysis (peak at 76 cM with LOD = 4.5), although failing to reach significance (Supplementary Fig. 5).

### Genotypic information coefficient (GIC)

**Fig. 5:**
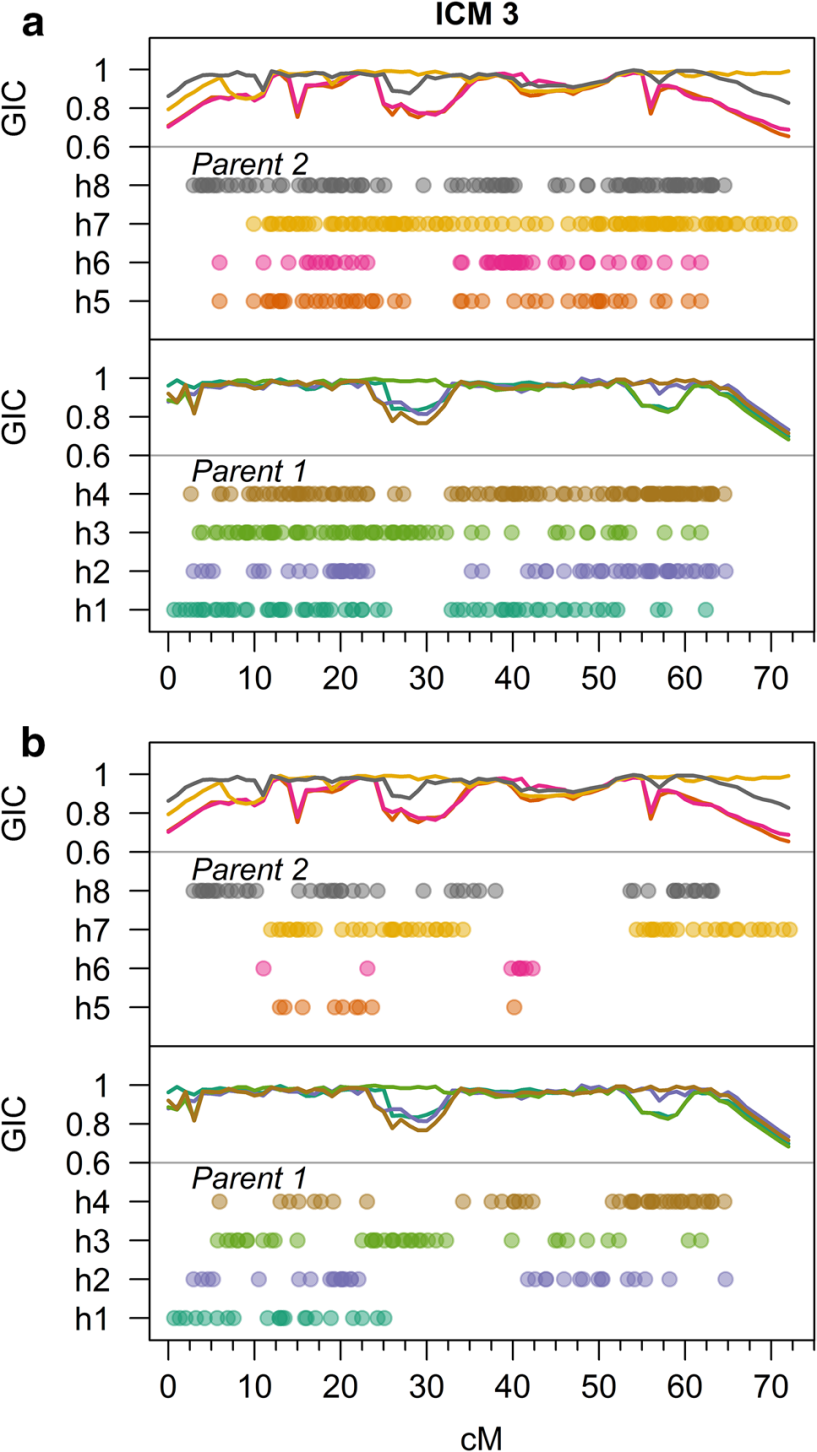
Example of the distribution of segregating marker alleles across the eight parental homologues on linkage group ICM 3, with the per-homologue genotypic information coefficient (GIC) plotted above. Lower sections show the four homologues of Parent 1 (h1–h4), while the upper sections show those of Parent 2 (h5–h8). GIC values were found to be in the range 0.6–1 (approximately) for both parents, with higher GIC values indicating greater amounts of genetic information for that homologue. Part **a** shows the marker allele distribution of all markers in their converted form (except for 1 × 3 markers which were (for the sake of this figure) converted to 1 × 1 to depict the segregating allele in parent 2 as simplex rather than triplex. In part **b**, all duplex occurrences have been removed (so that for example a 1 × 2 marker now appears as 1 × 0), to highlight homologue-specific marker alleles

The genotypic information coefficient gives a measure of the level of information provided by marker data on a scale from 0 to 1, with 0 corresponding to no information and 1 to full information (and is homologue specific in our formulation). When based on IBD probabilities, GIC can be thought of a measure of how certain we are about the inheritance of a parental homologue at a particular position, averaged across the population. Most GIC values exceeded 0.9, reflecting the dense marker coverage across all parental homologues. An example of the GIC profile for linkage group ICM 3 is shown in Fig. 5. The telomeric region of homologue h7 of parent 2 had additional marker coverage when compared to the other parental homologues (due to a 10-cM stretch of simplex × nulliplex markers which mapped there). The GIC content on all other seven homologues steadily decreases towards the telomere in this region, while for homologue h7 it remains close to 1. Dips in the GIC can also be seen to correspond to areas of lower marker density along the chromosome (Fig. 5), although a precise correspondence is difficult to ascertain as markers in duplex condition provide relatively balanced (albeit paired) inheritance information on all homologues of an autotetraploid (these are suppressed in Fig. 5b to display only homologue-specific marker alleles). The GIC profiles for all seven rose linkage groups are provided in Supplementary Fig. 6.

## Discussion

### Choice of QTL model

We detected a number of extra QTL using the one-stage analysis, which were not significant using the two-stage approach (for stem width and side shoots). However, we have no guarantee that these represent true QTL positions and not false-positive results. It would be useful to compare different approaches to modelling multi-environmental QTL in polyploids using simulated data, but this fell outside the scope of the present study. Regarding computation time, the two-stage QTL results shown in Fig. 2 (including permutation tests) took 1.3 h to generate; in comparison, the one-stage analysis took 9.5 h. However, this comparison is not entirely fair as the former analysis was performed on 6 cores, while the latter was run on a single core (due to problems encountered while attempting to run the lmer function in parallel). Overall, the one-stage approach appears to be slightly more powerful, while the two-stage analysis gave similar results while being arguably simpler to implement.

### Multi-environment QTL

QTL studies often report QTL without necessarily considering whether these QTL will be useful in practice. One of the more important considerations is whether the reported QTL are robust across environments or are only expressed in a specific environment. This is particularly relevant for modern rose breeding, for which the selection and production environments often differ (Gitonga et al. 2014). In this study, we analysed phenotypic data from three locations, two in Kenya and one in the Netherlands, with an extra set of phenotypes evaluated during both summer and winter seasons in the Netherlands. We were most interested in identifying QTL that are effective across both the selection and target environment. We found that a number of QTL were environment specific, such as the QTL for stem length present on linkage group ICM 4, but most QTL were identified in both the Kenyan and Dutch trial data (Fig. 3). The implication is that for the traits studied here, marker-assisted selection in a non-target environment should also produce rose cultivars that express the desired traits in the production environment. The previous analysis of Gitonga et al. (2014) reported high heritabilities for these traits across environments. Among the traits with the lowest reported heritabilities were chlorophyll content and number of side shoots (Gitonga et al. 2014), both traits for which only minor QTL were detected in the current analysis. These traits both showed a lower consistency across environments (Supplementary Fig. 1), as well as large genotype × environment (G × E) interaction effects in a previous study (Gitonga et al. 2014).

### Consistency with previous studies

Many of the traits examined in the current study have previously been mapped in other populations (mainly, but not exclusively at the diploid level). Table 3 summarises the main QTL identified by other groups for the morphological traits examined here. Attempting to compare results from previous studies relies on consistent linkage group numbering; the work of Spiller et al. (2011) has done much to improve this situation, but not all previously published maps were included in that study. In future, the recently published rose genome sequence will provide a standard reference map against which results can be compared (Hibrand-Saint Oyant et al. 2018).

#### Comparison to previous tetraploid studies

There were slight differences in our detection of QTL for stem prickles and those of a previous tetraploid study that used the same population and phenotypic data but different genotype data (Koning-Boucoiran et al. 2012). The main difference in these studies was the amount of marker data, which went from 469 mapped AFLP and SSR markers to 25,695 mapped SNP markers. This greater coverage probably enabled the detection of QTL on (parts of) linkage groups that were not covered by markers in the initial study (like the QTL peaks on ICM 4 and 6 that we detected). In the original maps, only 3 of the 8 parental homologues of linkage group 6 were identified, and there remains some uncertainty over the homologue assignment of linkage group 4. The ICM 2 QTL that was missed in our multi-environment analysis was detected in the single-environment scan (Fig. 3), which suggests this is not a robust QTL across environments.

Both Rajapakse et al. (2001) and Zhang et al. (2006) reported finding a QTL for petiole prickles in a tetraploid F_2_ mapping population on their linkage group “B7” (Table 3). Zhang (2006) provided the primer sequences of microsatellite markers Rw55D22 (GATCCGTTTAAGTAACCTTTCCACAAGGATTCTGATTTAT) and Rw5D11 (CAGATTCGCCGTAGCCCTTACATCCGAACCCCGACCTGAC) which were reportedly linked to this trait (Zhang et al. 2006). A BLASTn of these primer sequences on the *Rosa chinensis* Genome v1.0 chromosomes (Hibrand-Saint Oyant et al. 2018) using the online BLAST facility at https://www.rosaceae.org/blast produced hits on rose chromosome 6. Intriguingly, the marker Rw5D11 was previously used in genotyping the K5 mapping population (Koning-Boucoiran et al. 2012) and was mapped on LG 4, which would be consistent with our result of a single major QTL for petiole prickles on ICM 4 (Figs. 2, 3). However, this marker (Rw5D11) showed greatest linkage to simplex markers on ICM 6 (Bourke et al. (2017), Table S5: recombination frequency *r* ≤ 0.09 and LOD ≥ 20.4), consistent with the BLASTn results. Indeed, a more detailed look at the AFLP and SSR maps of Koning-Boucoiran et al. (2012) with the SNP maps of Bourke et al. (2017) shows that linkage groups A4-1, A4-2 and A4-3 should probably have been assigned to ICM 6, and A4-4 to ICM 4 (in other words, the homologues of linkage group A4 do not appear to form a single chromosomal linkage group). It is therefore unclear where the QTL for petiole prickles of Rajapakse et al. (2001) and Zhang et al. (2006) should be placed, on ICM 4 or ICM 6. These sorts of examples demonstrate the usefulness of a single reference sequence in allowing unambiguous comparisons between different results.

Despite these hurdles, we were able to confirm many of the previously reported QTL in the present study, as well as providing evidence for some previously unreported QTL for these important morphological traits (e.g. the QTL for stem prickles on ICM 6 and the QTL for petal number on ICM 2).

#### Comparison to previous diploid studies

In general, the results from diploid studies tally well with our results from a tetraploid analysis (e.g. Linde et al. (2006) or Debener and Mattiesch (1999)). Conversely, many of the QTL reported from the diploid 94/1 population by Yan et al. (2007) were not detected in this study [e.g. QTL for stem length and width on linkage groups ICM 1 and 5, or the four separate QTL detected for chlorophyll content (Table 3)]. This may suggest that either large differences in the genetic control of some morphological traits could exist between ploidy levels, or (more likely) that QTL for these traits segregated in their population while not in ours. Another possible cause is differences in the methodologies of QTL detection and particularly significance threshold setting, which may have led to either inflated Type I or Type II errors between their study and this one, respectively. As long as rose breeding and production continues to be performed at the tetraploid level, QTL studies at the tetraploid level continue to have clear advantages over diploid studies as was already discussed. Marker assays that have been identified at the diploid level may not necessarily perform well at the tetraploid level (e.g. due to unknown flanking SNPs or off-target hybridisation). Tetraploid offspring with desirable combinations of traits can be directly used in further breeding work (or may represent a finished variety), whereas there is less use for offspring of a diploid cross. Furthermore, although there are still many more tools available for diploid genetic analysis, those for tetraploid studies are becoming increasingly sophisticated and are helping to bridge the gap between the two ploidy levels. In order to be deployed in a breeding programme effectively, QTL should ideally be of large effect, stably expressed across environments, and effective irrespective of genetic background. In this study, we looked at stability across environments, but it could also be argued that our results also illustrate that these QTL are stable across genetic backgrounds as they have been detected in a number of independent populations both at the diploid and tetraploid level. The effect sizes of the QTL detected in this study were in general relatively modest (Table 1; Supplementary Fig. 7), although they may still be large enough to be of interest to rose breeders.

Finally, a number of possible candidate genes underlying key morphological traits such as prickle density (*TESTA TRANSPARENT GLABRA2* homologue on ICM 3) or petal number (*APETALA2/TOE* homologue on ICM 3) have recently been proposed (Hibrand-Saint Oyant et al. 2018). Interestingly, it appears that the number of prickles on the stem and petiole is regulated differently, as we found a major peak on ICM 4 for petiole prickles but no evidence for a role of the ICM 3 prickle density QTL. The ICM 3 peak for petal number probably also corresponds to the *APETELA2* locus, although the presence of a second peak on ICM 2 shows that work remains in fine-mapping these important QTL.

### IBD-based QTL analysis versus single-marker ANOVA

One of the principal advantages of QTL analyses using IBD probabilities in polyploids is that QTL detection is performed across all homologues simultaneously. Single-marker approaches rely on coupling linkage between marker alleles and QTL alleles, and may be less powerful due to “genotypic noise” from other homologues if the marker is not in complete coupling phase with the QTL alleles. This becomes even more important at higher ploidy levels where there are even more QTL configurations possible (van Geest et al. 2017), decreasing the likelihood of full coupling linkage between a QTL and a nearby marker. In this study, we performed both an IBD-based and a single-marker ANOVA QTL analysis. The results from both approaches were relatively consistent (as a comparison of Tables 1 and 2 shows), albeit with a much more detailed picture emerging from the IBD-based analysis.

Although we had assumed that IBD-based methods should generally be more powerful at detecting QTL, we found that both methods gave similar results (particularly if we consider the combined results from the one-stage and two-stage IBD-based analyses). However, apart from detection power, IBD-based analyses also offer the possibility to determine the most likely QTL segregation type and mode of action, which can only be guessed at using single-marker approaches (i.e. by looking at the segregation type of the most significant marker, Table 2).

The Bayesian information criterion (Schwarz 1978) has previously been shown to correctly identify the QTL model in polyploid QTL studies, with prediction accuracies of up to 90% for simplex QTL (details in Bourke et al. 2018). Phased QTL information could help improve the accuracy of marker-assisted selection, where breeders would no longer select individuals based on single-marker alleles, but on desirable combinations of parental haplotypes (through a combination of specific SNP alleles). Haplotype-assisted selection in polyploids has yet to be applied (to our knowledge) but would require much clearer QTL phasing than we were able to achieve in this study to be able to reliably construct a potential QTL-tagging haplotype marker (for all QTL peaks there were at least two plausible models within 6 BIC of the minima). The possible causes for the uncertainty are many—conflicts in genotype information, inaccurate linkage maps, more complex QTL types, poorly scored or confused phenotype data, etc. Despite this, our approach of saturating QTL regions with extra markers tended in general to increase the significance and proportion of explained variances at QTL positions (Table 1), with a single exception that may have been due to a sub-optimal phasing solution returned by TetraOrigin [which is a stochastic rather than deterministic procedure (Zheng et al. 2016)]. High genotypic information coefficients around QTL positions on the relevant homologues have been shown to be necessary for the accurate and clear detection of QTL as well as the precise estimation of their location and phase (Bourke et al. 2018) and need to be taken into account if QTL studies in polyploids are to deliver applicable results. Our visualisations of allelic effects around the QTL peak positions will also be useful in a breeding context, allowing a breeder to quickly identify desirable haplotypes within the population, as well as critically assess the BIC results. However, translating these haplotypes into selectable markers that are applicable in more diverse germplasm will require either validation experiments, or preferably the development of methods and tools that use multiple (related) populations rather than single *F*_1_ crosses (i.e. simultaneous detection and validation within one analysis).

## Concluding remarks

In this study, we applied recently developed methods to help unravel the genetic architecture underlying a number of important morphological traits in tetraploid rose. Most of the detected QTL were found to be robust across environments, suggesting that selection for these traits can successfully be performed in locations other than the production site. Apart from identifying and confirming an important set of QTL at the tetraploid level, our work helps pave the way towards haplotype-assisted selection methods in the future.

## Author contribution statement

PMB performed the final data analysis, developed the methodology and wrote the manuscript; VWG conceived of the study, collected the phenotypic data and performed the initial data curation; REV and CM helped develop the methodology and edited the manuscript; RGFV and FK participated in coordination and supervision and helped revise the manuscript. All authors read and approved the final manuscript.

## Electronic supplementary material

Below is the link to the electronic supplementary material.

Supplementary material 1 (PDF 5736 kb)
