# Supplementary material for: Multi-environment QTL analysis of plant and flower morphological traits in tetraploid rose
Source: Theor Appl Genet. 2018 Jun 30;131(10):2055–69. doi: 10.1007/s00122-018-3132-4 (PMC6154034; doi:10.1007/s00122-018-3132-4)
Supplement: Supplementary file 1 — Supplementary material 1 (PDF 5736 kb) [file 122_2018_3132_MOESM1_ESM.pdf]

Supplementary material

Supplementary Table 1 Description of horticultural traits evaluated and scored in the tetraploid K5 population. Fuller descriptions can be found in Gitonga et al. (2014).

**Trait**	**Description**
Date of Bending	The bending dates were given the following numerical scores: 1 = 29, 2 = 32, 3 = 37, 4 = 39, and 5 = 44 days after planting.
Plant height	Height (cm) from the rim of the pot to the highest apical bud before bending.
Plant vigour	Plants ranked on a scale of 1-5 based on their height, number of stems, number of leaves and branching present before bending; 1 = low vigour, 5 = very vigorous.
Chlorophyll content	The chlorophyll content (mg/l) of the first fully formed, uniform green coloured leaves from the top, using a portable fluorimeter (PAM-2001) Walz, Effeltich Germany.
Number of Petals	The number of petals counted when the flower was in full bloom
Prickles on petiole	The number of prickles on the petioles that are formed between the 4th and 6th nodes
Prickles on stem	The number of prickles between the 4th and 6th nodes on the main stem
Stem length	Length (cm) from the floral tube to the shoot base
Side shoots	The number of side shoots on the whole stem
Stem width	Diameter (mm) of the stem at middle of the 2nd and 3rd internodes from shoot base

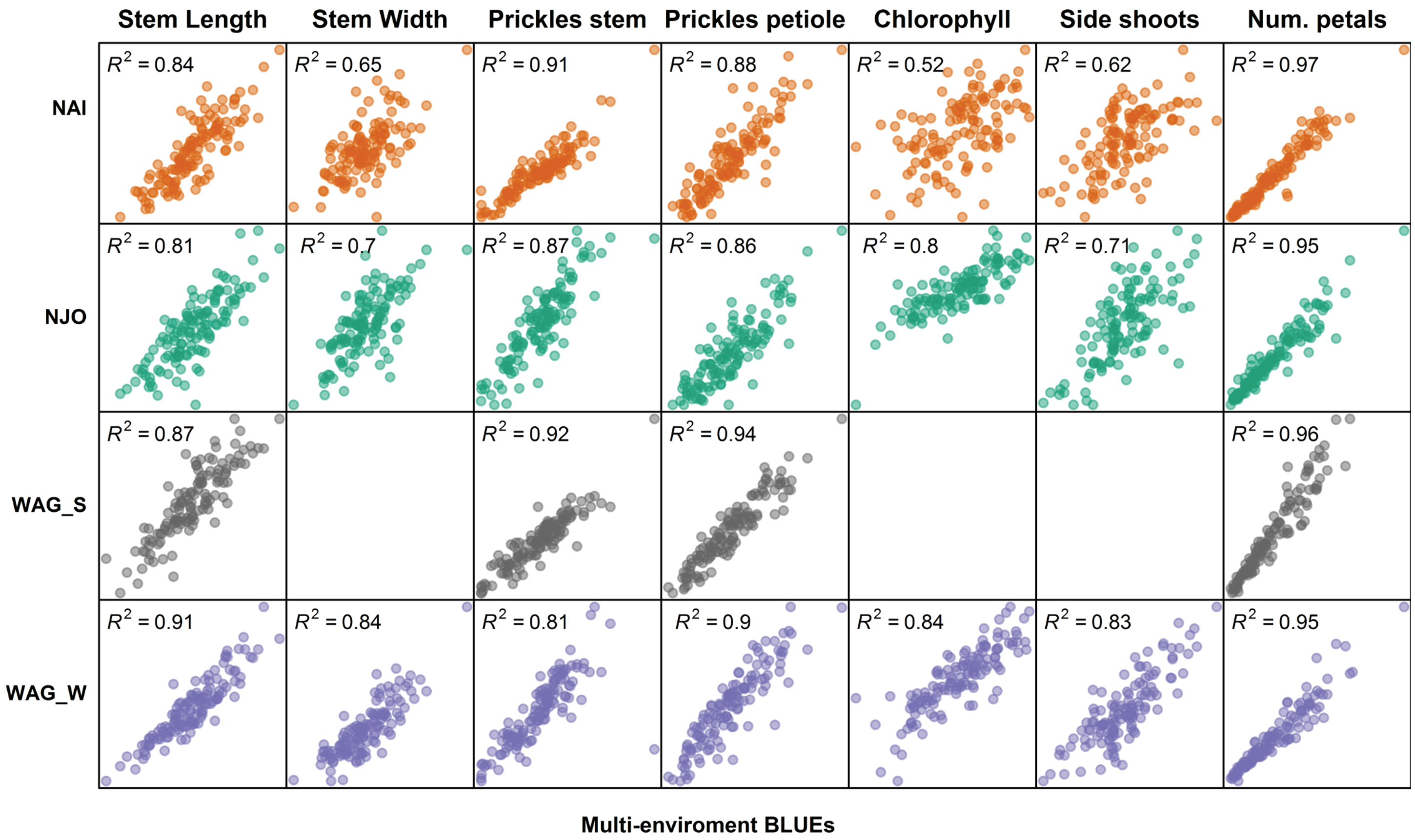

Supplementary Figure 1. Comparison between single environment and multi-environment Best Linear Unbiased Estimates (BLUEs) for each of the seven morphological traits recorded in this study.

The four environments are shown on the left-hand margin, namely Nairobi (NAI), Njoro (NJO), Wageningen summer (WAG_S) and Wageningen winter (WAG_W). The squared Pearson correlation coefficients (R^2) are printed for each comparison. Multi-environment BLUEs (x-axis) are equal across the four environmental comparisons.

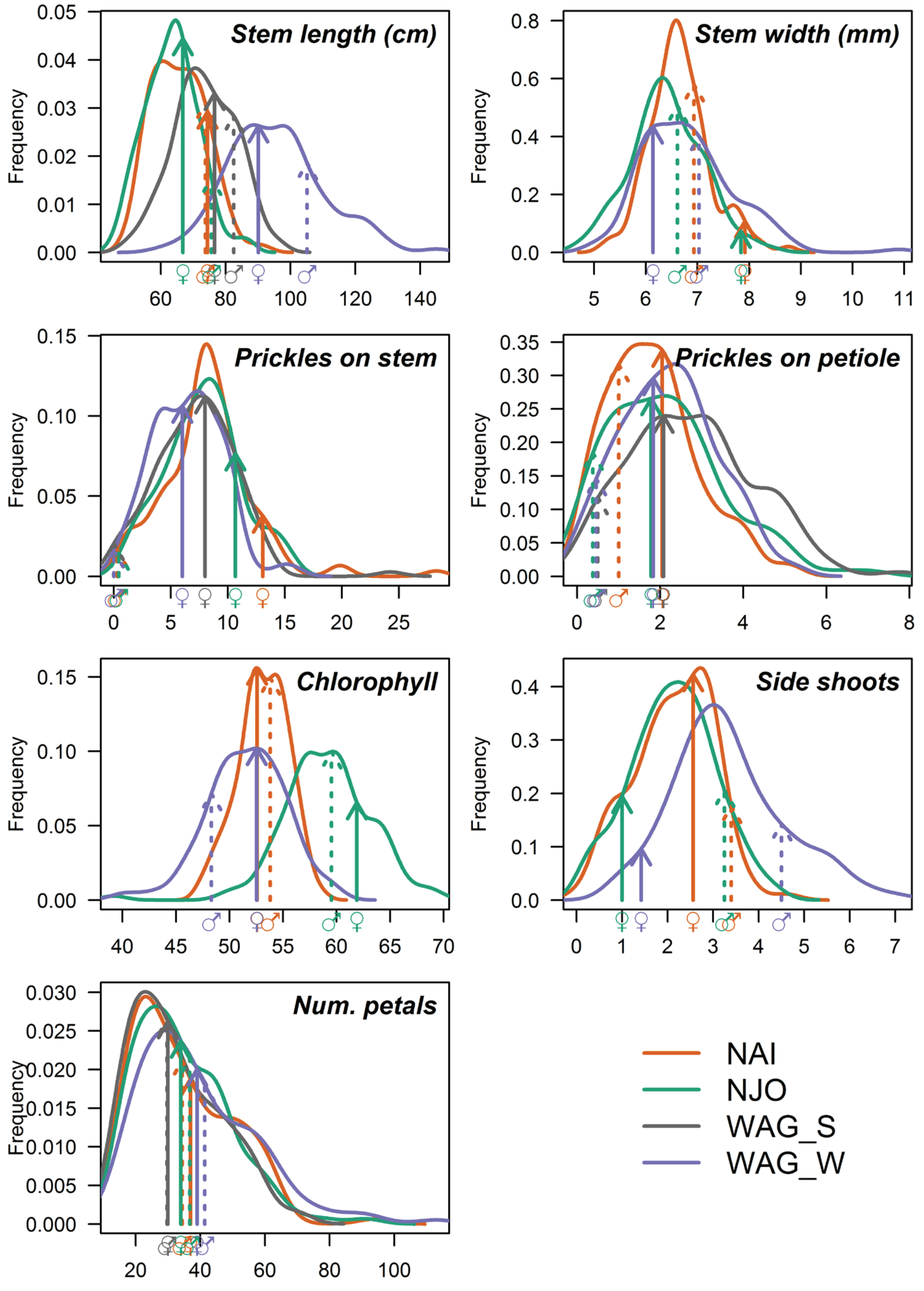

Supplementary Figure 2. Distribution of rose morphological trait values over four different environments (Nairobi (NAI), Njoro (NJO), Wageningen summer (WAG_S) and Wageningen winter (WAG_W)).

Mean parental values are shown as either solid lines (maternal mean, ♀) or dashed lines (paternal mean, ♂).

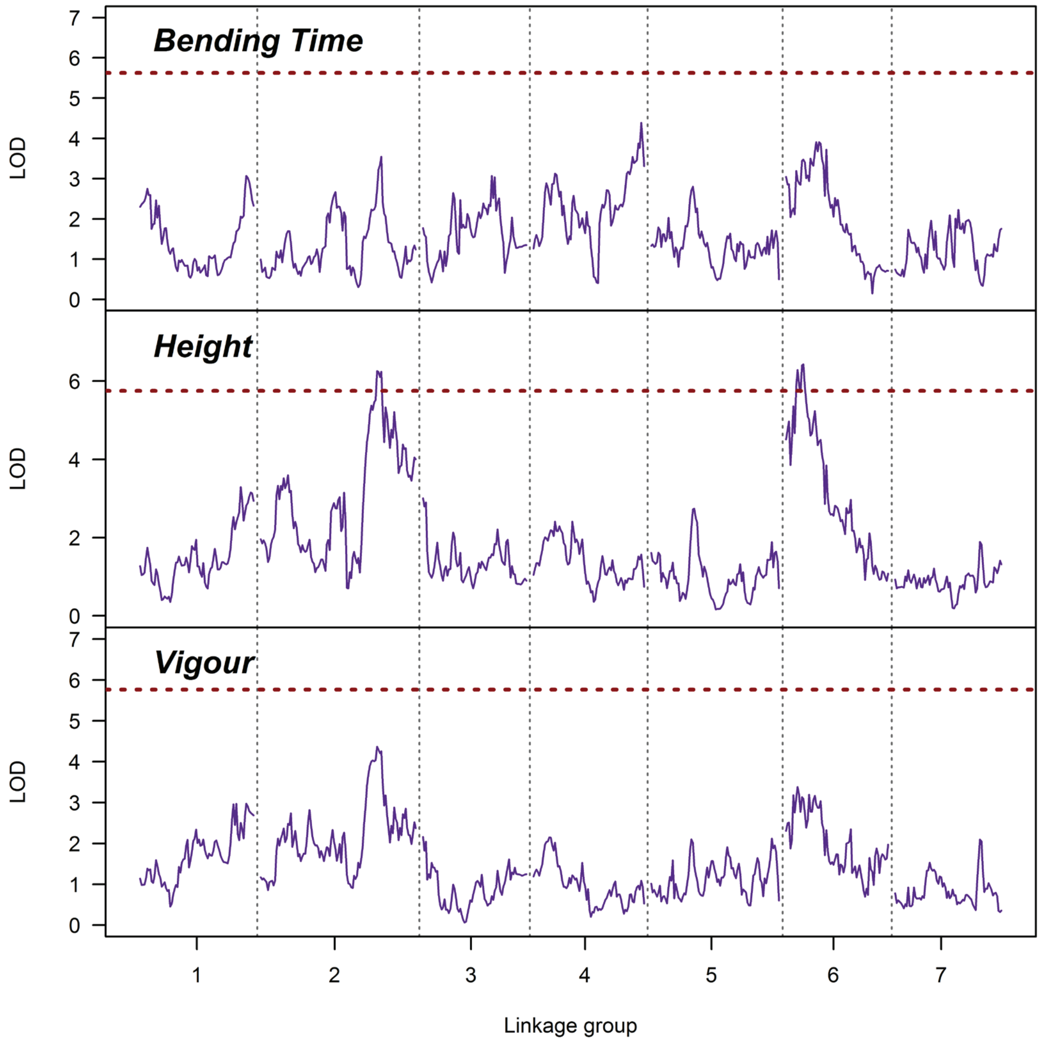

Supplementary Figure 3. Single-environment QTL analysis results for the traits bending time, plant height and plant vigour, assessed in Wageningen (WAG_S) only (using the two-stage approach).

Significance thresholds (as determined by permutation tests) are shown as dashed lines

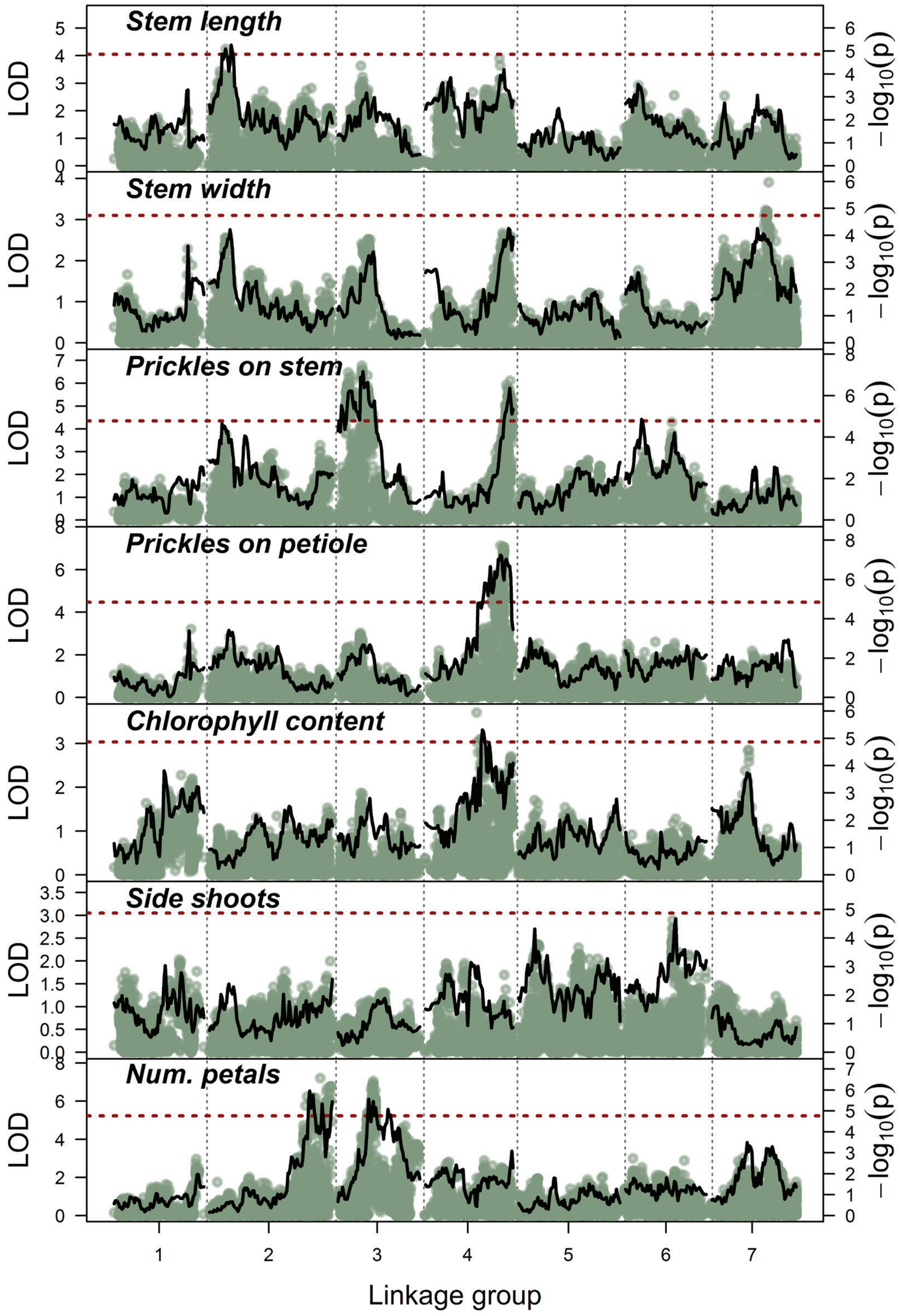

Supplementary Figure 4. Comparison between two-stage IBD-based QTL analysis results (black line) with a single-marker ANOVA results (green dots) for the seven morphological traits studied.

Significance thresholds (as determined by permutation tests) are shown as red dashed lines (plot data was rescaled so that these lines overlap). Significance values for the IBD-based analysis are given on the left-hand axes (LOD scores) with the ANOVA significance values on the right-hand axes ($-\log_{10}(p)$ values).

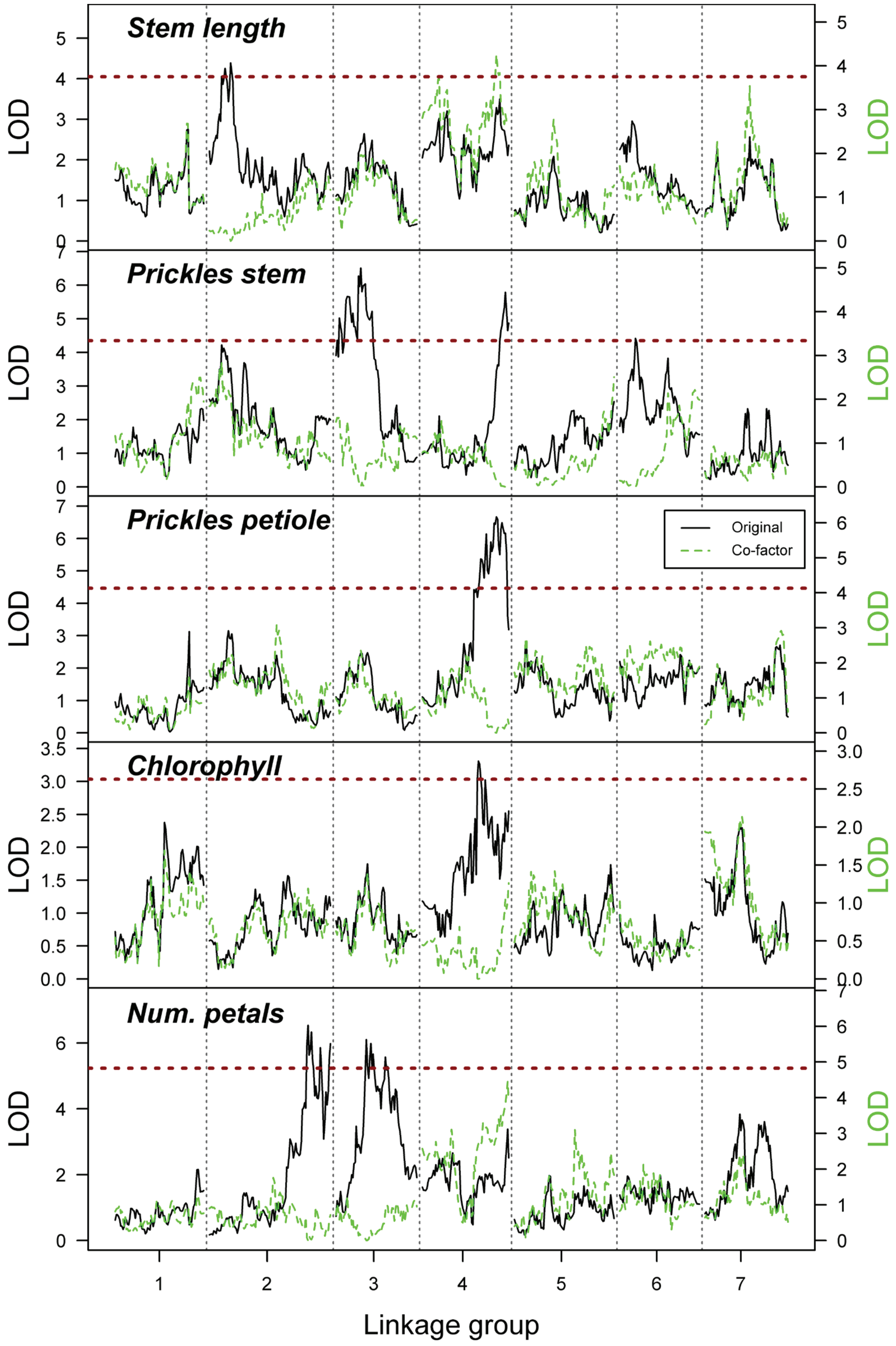

Supplementary Figure 5. Genetic co-factor analysis (using the two-stage approach for QTL detection).

For each of the five traits for which QTL were detected, the QTL peak position(s) were included as co-variates in a subsequent re-analysis (green dashed lines). Only for the trait stem length did this result in the detection of a "novel" QTL on linkage group ICM 4. The right-hand y-axis LOD scale corresponds to the co-factor analysis (green dashed lines) while the ordinary y-axis LOD scale corresponds to the original QTL analysis (black solid line). Significance thresholds as determined by permutation tests (N = 1000, α = 0.05) shown as a red dashed line (separate thresholds were calculated for both analyses).

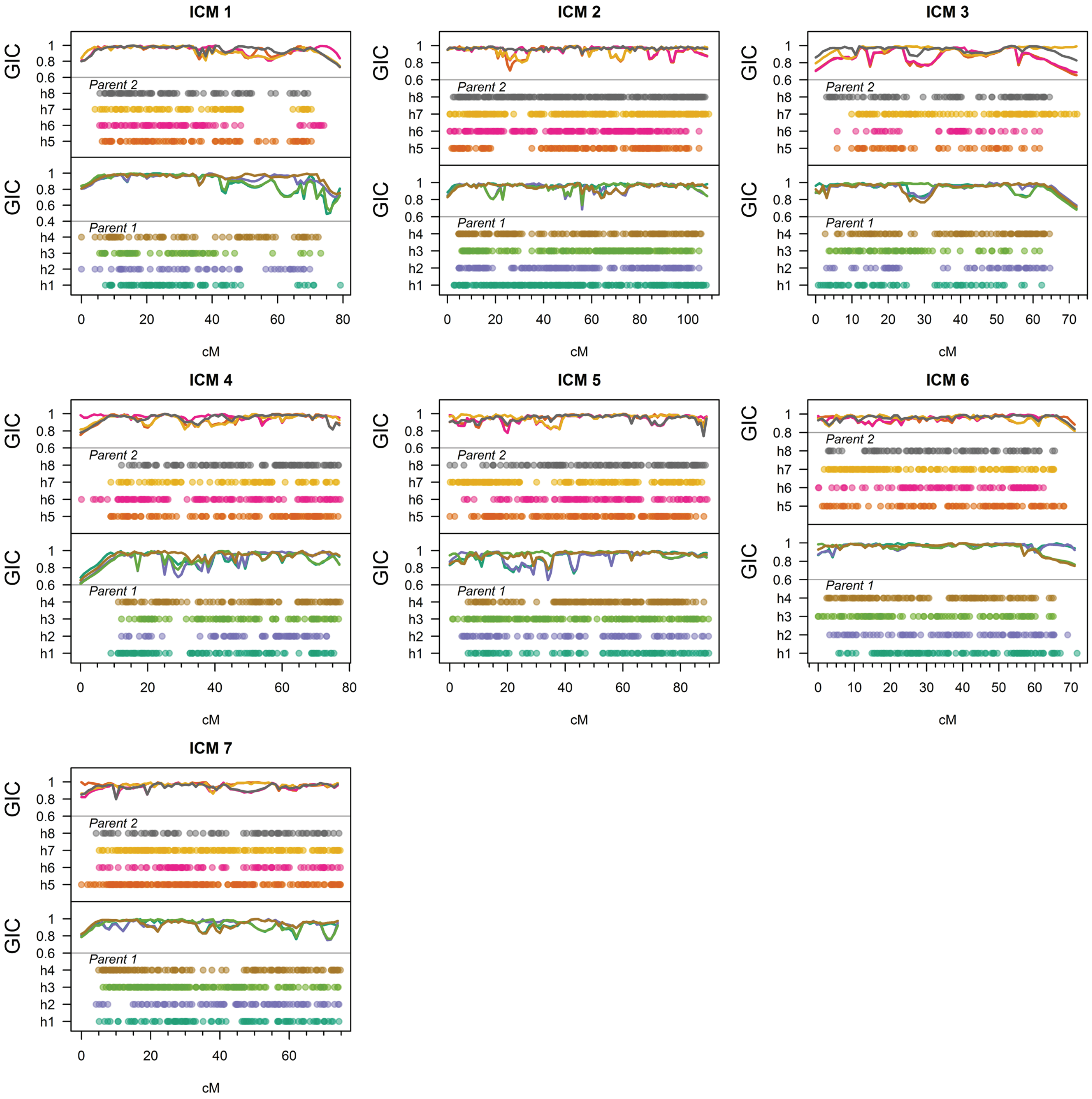

Supplementary Figure 6. Genotypic Information Coefficient (GIC) plots and segregating marker alleles for rose linkage groups 1 – 7, visualising the information content per homologue of the IBD probabilities used in the initial QTL scan.

Marker allele distributions for Parent 1 on homologues h1 – h4 are shown in the lower section, with marker allele distributions for Parent 2 (homologues h5 – h8) shown in the upper section. GIC values were found to be in the range 0.6 – 1 (approximately) for both parents, with higher GIC values indicating greater amounts of genetic information for that homologue. The colouring scheme of the GIC per homologue and the marker distribution on that homologue are identical. 1x3 markers were temporarily converted to 1x1 to highlight only a single segregating allele in parent 2.

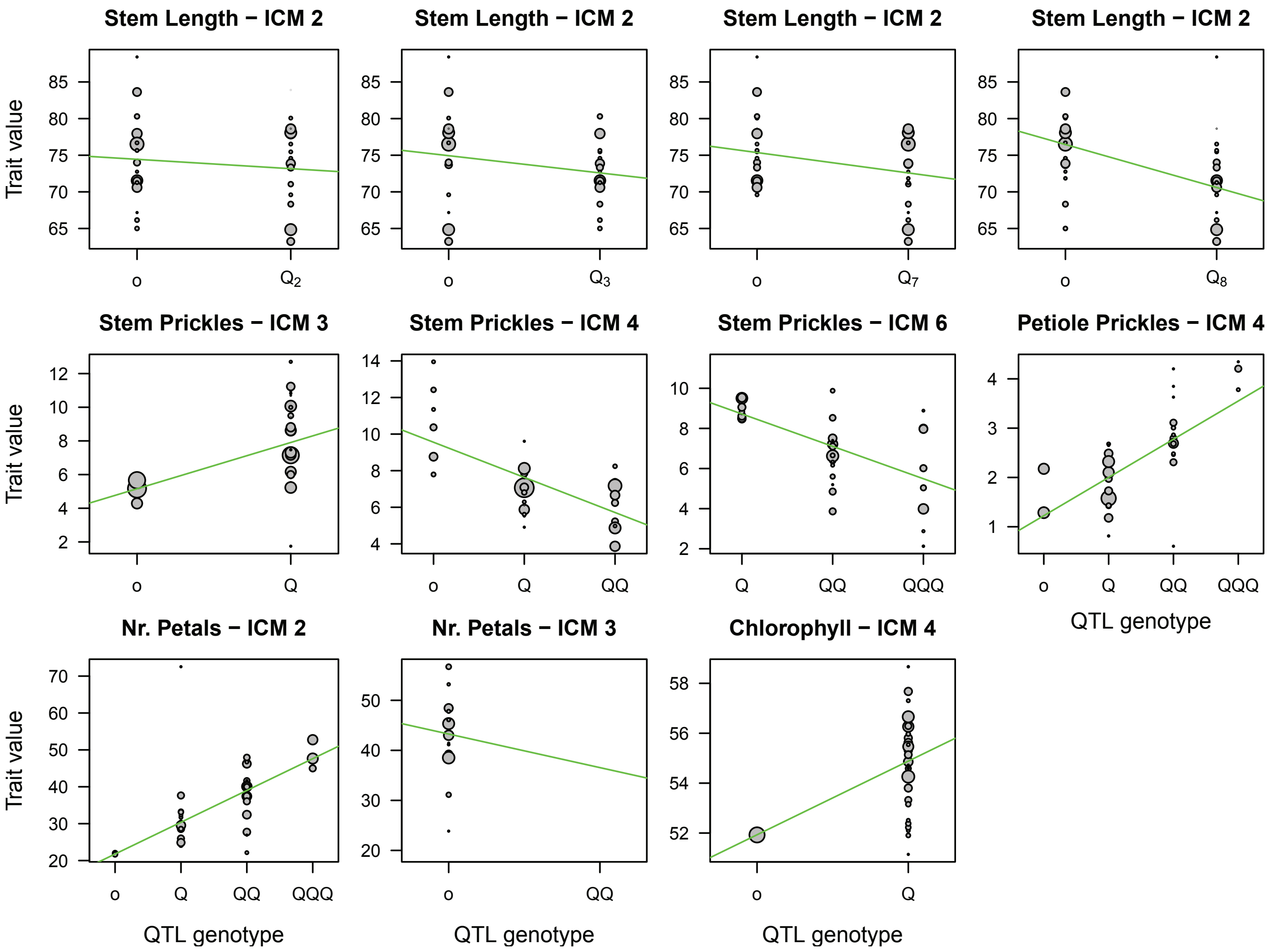

Supplementary Figure 7. Mean trait values per QTL genotype class at each of the detected QTL peaks.

The size of each point corresponds to the summed offspring probabilities for that class (approximating the number of offspring in each of the 36 genotype classes), with the fitted regression line, weighted by these summed probabilities, shown in green.
